# What factors are associated with anaemia in pregnancy among Nigerian women? Evidence from a national survey

**DOI:** 10.4314/ahs.v23i1.39

**Published:** 2023-03

**Authors:** Emmanuel Olusola Adeyemi, Temitope Olumuyiwa Ojo, Megan Quinn, Bill Brooks, Oluwabunmi Anuoluwapo Oke

**Affiliations:** 1 East Tennessee State University, Department of Biostatistics and Epidemiology; 2 Obafemi Awolowo University College of Health Sciences, Department of Community Health; 3 Heatherwood Hospital, Department of Surgery

**Keywords:** Determinants, Anaemia, Pregnancy, Nigerian women

## Abstract

**Background:**

Anaemia in pregnancy remains a severe public health problem in sub-Saharan African countries including Nigeria.

**Objectives:**

To assess factors associated with anaemia in pregnancy among Nigerian women.

**Methods:**

A secondary analysis of the 2018 Nigeria demographic health survey was conducted to determine the predictors of anaemia among Nigerian pregnant women (N=1522). SAS 9.4 was used for the analysis.

**Results:**

The prevalence of anaemia in pregnancy was 61.1%. On multivariable logistic regression analysis, women in the North-central (AOR=2.52, CI=1.46-4.35) and South-south (AOR=2.21, CI=1.06-4.59) had increased odds of anaemia in pregnancy, compared to those in the Northwest. Women with no education (AOR=2.38, CI=1.28-4.44), primary education (AOR=3.06, CI=1.58-5.96) and secondary education (AOR=1.75, CI=1.04-2.94) had increased odds of anaemia in pregnancy compared to women with teriary education. Also, women not in marital union had increased odds of anaemia in pregnancy compared to women in a union (AOR=2.56, CI=1.15-5.72). Women in the second (AOR=2.42, CI=1.79-3.29) and third trimesters of pregnancy (AOR=2.83, CI=2.07-3.89) had increased odds of anaemia.

**Conclusion:**

These findings are important for the control of anemia among pregnant Nigerian women. Women in the Northcentral and Southsouth zones are particularly at risk for anaemia in pregnancy and should receive special attention during antenatal care.

## Introduction

Anaemia is a common complication of pregnancy worldwide because of the physiological changes that occur during pregnancy.^1^ Worldwide, the prevalence of anaemia in pregnancy is estimated at 38%.^2^ The prevalence is typically higher in women in developing countries compared to their counterparts in developed countries due to poor diet, cultural beliefs, lack of education, poor access to efficient health care.^3^ In many sub-Saharan African countries, the prevalence of anaemia in pregnancy is a severe public health problem according to the WHOs classification of anaemia in populations since the levels are higher than 5%.

Majority (75%) of anaemia in pregnancy is attributable to iron deficiency.^1^ This is particularly true in developing countries where premenopausal women generally have lower serum iron due to chronic loss of blood from menstruation which prevents building up of iron stores needed for hemopoiesis.^4^ For instance, as at 2016, the prevalence of anaemia in Nigerian pregnant women was estimated to be 58% 5 and this may be explained by iron deficiency due to poor nutritional intake, chronic blood loss from menstruation, parasitic infestation like hookworm and malaria in pregnancy which are common in developing countries like Nigeria. Other factors that may be associated with higher prevalence of anaemia among pregnant women in developing countries include socio-economic and demographic characteristics like low economic status 6, poor educational attainment 7 and higher parity 8.

With the high burden of anaemia in pregnancy in Nigeria and the attendant complications on mother and child (fetal growth restriction, infant mortality, postpartum depression, maternal heart failure, maternal mortality etc.,) that has been associated with anaemia in pregnancy, there is the need to identify the sociodemographic and obstetrical and gynaecological factors associated with this public health problem among Nigerian women using a nationally representative sample. This study seeks to address this knowledge gap in the literature and provide information that may be useful in planning interventions aimed at reducing the burden of anaemia in pregnancy in Nigeria.

## Methods

### Study design

This is a cross-sectional study that used a secondary data from the 2018 Nigerian demographic health survey (DHS) to estimate the prevalence and predictors of anaemia among pregnant women in Nigeria. Our study population comprised pregnant women in the survey (N=1522).^9^ For stratification, each of the 36 states of the federation and the Federal Capital Territory was divided into urban and rural areas. An urban area being any locality with more than a minimum population of 20,000 and rural areas being a locality with less than a population of 20,000.^9^ In the first stage, 1400 census enumeration areas (EA) were selected with probability of selection being proportional to the EA size (number of households in the EA). [9] In the second stage, the sample frame was a listing of household in the 1400 census enumeration areas, and a fixed number of 30 households was selected in every cluster through equal probability systematic sampling, resulting in a selection of about 42,000 households for the survey. 9 A tablet was used for the household listing and a computer program was used for random selection of households. Only selected households were interviewed. Haemoglobin levels to determine anaemia was done by obtaining small volume of capillary blood in the fingers.^10^ The finger was warmed by rubbing the hands and cleaned with alcohol before making a finger prick through sterile, retractable lancet. The first two free-flowing drops of blood were wiped away with a sterile piece of gauze while the third drop was sampled with a microcuvette.^10^ HemoCue system (HemoCue 201+ or HemoCue 301+) was then used to determine haemoglobin concentration.^10^ The fourth or fifth drop of blood was occasionally used for haemoglobin measurements while other parameters were being measured. Haemoglobin levels were adjusted for both altitude and cigarette smoking.^10^ A pregnant woman was defined as anemic if adjusted haemoglobin levels were less than 11g/dl. 11

### Data analysis

The outcome variable was anaemia status while explored predictors included age group, region of residence, rurality, highest educational level, religion, ethnicity, marital status, wealth index, trimester of pregnancy, parity, ever wanted the pregnancy, and intention to use contraceptive. The study analyzed weighted percentages of the variables and conducted comparisons of outcome groups based on the predictor variables using Chi-square. Univariable and multivariable logistic regression was used to explore the strength of relationship between the outcome variable and the predictor variables. Only variables with P-value≤0.05 in the bivariate analysis were considered for inclusion in the regression models. The P-value for both univariable and multivariable analysis was obtained from Wald Chi-square. The goodness of fit of the model was examined using Hosmer-Lemeshow test and the predictive capability of the model was examined using the concordance index (C-statistic). SAS 9.4 was used for the statistical analysis.

### Ethical consideration

Ethics approval to conduct the NDHS 2018 was obtained from the National Health Research Ethics Committee (NHREC) of the Federal Ministry of Health, Nigeria. Approval to download and analyse the dataset was obtained from the Demographic and Health Surveys (DHS) programme.

## Results

### Prevalence of anaemia in pregnancy among Nigerian women

**Figure 1:**
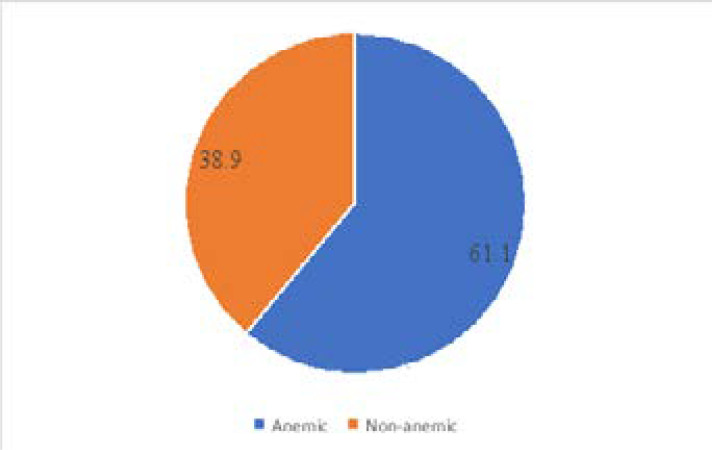
Prevalence of anaemia in pregnancy among Nigerian women.

As shown in figure 1, the prevalence of anaemia in pregnancy among Nigerian women is estimated at 61.1% (N=1522).

### Demographic characteristics of respondents

Majority (84.3%) of the respondents were aged 20 to 39 years while 69% of the respondents were resident in the North with over a third of the respondent (36.4%) being resident in the country's Northwest. Majority (61.1%) of the respondents were resident in the rural areas while 43.8% of respondents had no formal education. See table 1 for distribution of other patients' demographic characteristics.

**Table 1:** Sociodemographic characteristics of respondents. *(N=1522)*

Characteristics	Percentage
**Sociodemographic**	
**Age group**	
15–19	10.5
20–39	84.3
≥40	5.2
**Region of residence**	
Northcentral	14.7
Northeast	17.8
Northwest	36.4
Southeast	10.1
Southsouth	8.4
Southwest	12.4
**Rurality of residence**	
Rural	61.1
Urban	38.9
**Highest education level**	
No education	43.8
Primary	13.4
Secondary	35.0
Tertiary	7.8
**Religion**	
Christianity	37.7
Islam	61.7
Others	0.5
**Ethnicity**	
Hausa/Fulani	45.6
Igbo	12.3
Yoruba	30.7
Minorities	11.4
**Marital Status**	
In a union	97.3
Not in a union	2.7
**Wealth Index**	
Poorest	21.1
Poorer	22.5
Middle	22.1
Richer	18.7
Richest	15.5

### Obstetric and gynaecologic characteristics of respondents

The trimester of pregnancy was almost equally distributed with 30.9%, 35.3% and 33.8% being in the first, second and third trimester, respectively. See table 2 for distribution of other obstetric and gynaecologic characteristics.

**Table 2:** Obstetrics and gynaecological characteristics of respondents. *(N=1522)*

Characteristics	Percentage
**Trimester of pregnancy**	
First trimester	30.9
Second trimester	35.3
Third trimester	33.8
**Parity**	
0	14.0
1	19.1
2–3	17.2
≥4	49.4
**Ever wanted current pregnancy**	
Yes	97.2
No	2.8
**Intention to use contraceptive**	
Intention to use	51.7
No intention to use	48.3

### Bivariate analysis

On comparing of the outcome groups (anaemia vs no anaemia) with the categories of the independent variables, the following variables showed significant difference between categories: region of residence, rurality, highest educational level, ethnicity, wealth index, trimester, marital status. See table 3 and 4 for the level of significance for each of the variables.

**Table 3:** Association between respondents' characteristics and anaemia status. (N=1522)

Characteristics	Percentage Anaemic	Non-anaemic	P-value
**Age group**			
15–19	11.0	9.9	0.2321
20–29	84.6	83.8	
30–49	4.5	6.3	
**Region**			
Northcentral	16.7	11.6	**0.0017**
Northeast	16.3	20.2	
Northwest	35.7	37.5	
Southeast	11.8	7.5	
Southsouth	8.1	8.9	
Southwest	11.3	14.2	
**Rurality**			
Rural	64.5	55.7	**0.0006**
Urban	35.5	44.3	
**Educational level**			
None	46.0	40.5	**<0.0001**
Primary	15.4	10.3	
Secondary	33.3	37.8	
Tertiary	5.4	11.5	
**Religion**			
Christianity	36.5	39.6	0.2199
Islam	63.1	59.6	
Others	0.4	0.8	
**Ethnicity**			
Hausa/Fulani	46.1	44.8	**0.0096**
Igbo	14.2	9.2	
Minority	28.9	33.7	
Yoruba	10.8	12.4	
**Marital Status**			
In a union	96.5	98.6	**0.0123**
Not in a union	3.5	1.4	
**Wealth Index**			
Poorest	23.0	18.2	**0.0004**
Poorer	24.2	19.9	
Middle	22.7	21.2	
Richer	17.1	21.2	
Richest	13.0	19.5	

Significant P-values are in bold.

**Table 4:** Association between respondents' obstetrics and gynaecology characteristics and anaemia status

Characteristics	Percentage		P-value
**Trimester**	**Anaemic**	**Non-anaemic**	
First	22.7	44.0	**<0.0001**
Second	39.0	29.5	
Third	38.4	26.6	
**Parity**			
0	14.5	13.2	0.3997
1	17.9	21.0	
2–3	31.6	32.2	
≥4	36.1	33.6	
**Ever wanted** **current pregnancy**			
Yes	96.7	97.9	0.1731
No	3.3	2.1	
**Intention to use** **contraceptive**			
Yes	50.4	53.8	0.1925
No	49.6	46.2	

Significant P-values are in bold.

### Factors associated with anaemia in pregnancy among Nigerian women

#### Sociodemographic factors

A univariable logistic regression to predict the effects of each of the sociodemographic variables on anaemia in pregnancy showed that pregnant women resident in the Northcentral had 51% (AOR=1.51, CI=1.05-2.17, P=0.0136) increased odds of anaemia compared to their counterparts in the Northwest. See table 5 for factors associated with anaemia in pregnancy.

**Table 5:** Factors associated with anaemia in pregnancy among Nigerian women

Characteristics	Crude OR 95% CI)	P-value	Adjusted OR (95% CI)	P-value
**Region**				
North Central	1.51 (1.05–2.17)	**0.0136**	2.52 (1.46–4.35)	**0.0125**
Northeast	0.85 (0.60–1.20)		1.11 (0.73–1.69)	
Northwest	1 (ref)		1 (ref)	
Southeast	1.65 (1.05–2.59)		1.71 (0.52–5.69)	
Southsouth	0.96 (0.57 -1.62)		2.21 (1.06 -4.59)	
Southwest	0.83 (0.50 -1.38)		1.57 (0.63 -3.90)	
**Rurality**				
Rural	1.44 (1.10–1.90)	**0.0089**	1.29 (0.94–1.78)	**0.1204**
Urban	1 (ref)		1 (ref)	
**Ethnicity**				
Hausa/Fulani	1.20 (0.90–1.61)	**0.0434**	1.54 (0.97–2.44)	0.0657
Igbo	1.81 (1.18–2.78)		2.71 (0.93–7.84)	
Minority	1 (ref)		1 (ref)	
Yoruba	1.01 (0.65–1.58)		1.53 (0.71–3.31)	
**Highest** **educational level**				
None	2.41 (1.53–3.78)	**0.0002**	2.38 (1.28–4.44)	**0.0114**
Primary	3.17 (1.84–5.45)		3.06 (1.58–5.96)	
Secondary	1.87 (1.17–2.98)		1.75 (1.04–2.94)	
Tertiary	1 (ref)		1 (ref)	
**Marital status**				
In a union	1 (ref)	**0.0207**	1 (ref)	**0.0220**
Not in a union	2.59 (1.16–5.81)		2.56 (1.15–5.72)	
**Wealth index**				
Poorest	1.89 (1.22–2.94)	**0.0202**	1.49 (0.78–2.83)	0.5489
Poorer	1.83 (1.19–2.83)		1.40 (0.78–2.52)	
Middle	1.61 (1.03–2.51)		1.10 (0.65–1.88)	
Richer	1.21 (0.74–1.97)		1.02 (0.58–1.78)	
Richest	1 (ref)		1 (ref)	
**Trimester**				
First	1 (ref)	**<0.0001**	1 (ref)	**<0.0001**
Second	2.56 (1.90–3.46)		2.42 (1.79–3.29)	
Third	2.80 (2.05–3.82)		2.83 (2.06–3.89)	

Significant P-values are in bold. OR=Odds Ratio. CI= Confidence Interval.

#### Obstetric and gynaecologic factors

The higher the trimester of pregnancy, the higher the odds of developing anaemia in pregnancy as women in the second trimester had 156% (AOR=2.56, CI=1.90-3.46, P<0.0001) increased odds of anaemia compared to women in first trimester, while women in the third trimester had 180% ((AOR=2.80, CI=2.05-3.82, P<0.0001) increased odds of developing anaemia compared to women in their first trimester. The woman's parity was not significantly associated with anaemia in pregnancy.

On adjusting for other obstetric, gynaecologic and sociodemographic variables (region of residence, rurality, ethnicity, wealth index, highest educational level, and marital status), trimester of pregnancy remains significant with women in the second trimester of pregnancy having 142% (AOR=2.42, CI=1.79-3.29, P<0.0001) increased odds of anaemia and women in the third trimester having 183% (AOR=2.83, CI=2.07-3.89, P<0.0001) increased odds of anaemia compared to women in their first trimester of pregnancy. See table 5 below for the effect of other obstetric and gynaecologic variables.

### Diagnostics of multivariable logistic regression model

The Hosmer-Lemeshow goodness of fit test chi-square was 0.0764 and the predictive capability (C-Statistic) was 66.0%.

## Discussion

This study assessed the prevalence and sociodemographic factors associated with anaemia in pregnancy among a nationally representative sample of Nigerian women. Although there has been increasing antenatal care attendance and administration of routine supplements among pregnant Nigerian women, the prevalence of anaemia in pregnancy remains high with a prevalence of 61% reported in this study. A 2016 World Bank Report had shown a 58.5% prevalence.^5^ Similarly, a higher prevalence of greater than 40% has been reported in the West African subregion and in other developing nations of the world like India. 8,12 The high prevalence reported in this study is a sharp contrast to the lower prevalence seen in developed countries.^13^ This difference can be attributed to better socioeconomic development, higher standards of living, better access and utilization of health care as well as higher literacy rates in developed countries compared to developing countries.

In the multivariable model, this study identified the following sociodemographic predictors: region of residence, highest education level, and marital status. The trimester of pregnancy was the only obstetric predictor of anaemia. A recent survey by the National Bureau of Statistics in Nigeria ranked regions in terms of prevalence of poverty with the following result: Northeast (71.86%), Northwest (64.84%), Northcentral (42.70%), Southeast (42.44%), Southsouth (21.28%), and Southwest (12.12%).^14^ In addition to the higher poverty rates in the Northeast and Northwest, these regions are largely plagued by insecurity and high level of illiteracy. The combination of these unfavourable social determinants of health may suggest that these regions would be more predisposed to anaemia in pregnancy, but our findings indicate that women in the Northcentral and Southsouth were more likely to have anaemia in pregnancy.

This study also showed that the educational attainment of a woman is a significant predictor of anaemia among pregnant women in Nigeria. This is not unexpected, given that highly educated women are more likely to have other favourable social determinants of health like access to health services, employment, higher family income, healthy housing, and they can easily follow through on the recommendations of their doctors or other health workers. This finding of association of education with anaemia has also been reported in another study in Northern Nigeria and in studies outside Nigeria. 7, 15

Our study also showed the reduced odds of anaemia among women in a marital union (married or living with a partner) compared to the women who were not in a union (single/divorced/widowed/ separated). This finding can be explained by the better social support that is available to pregnant women in a union. It would also seem that pregnant women in a union, especially those who have their own businesses or are employed, would have more economic resources at their disposal since they will be combining resources with their husbands who are more likely to be the breadwinner of the family in a typical Nigerian family setting. This finding of marital status being a significant predictor of anaemia in pregnancy was also corroborated by a study in Northern Nigeria. 7 This finding also has implications for preventing anaemia in pregnant women as interventions such as supplementation and nutritional counseling should be intensified among pregnant women not in a union.

The trimester of pregnancy is also another predictor of anaemia in pregnancy and there seems to be a dose-response relationship between the trimester of pregnancy and anaemia, with odds of developing anaemia being lowest in the first trimester and sharply rises in the second trimester with a further marginal rise by the third trimester. A retrospective study in China had shown higher incidence of anaemia as women progress through the trimesters. 16

A major strength of this study is the use of a national survey with a relatively large sample size that is representative of the country. Therefore, we believe this study provides generalizable findings to the Nigerian population, that policy makers and program planners can use to design intervention to address anaemia among pregnant women. A limitation of this study is its cross-sectional nature which makes us unable to establish the temporal relationship between the predictor variables and anaemia, therefore, we cannot categorically establish the cause-and-effect relationship between these variables and anaemia. Another limitation of this study is that we could only adjust for variables whose data were collected in the survey, meanwhile there are other variables like malaria, infection, nutritional intake, and other factors that are also important, but whose data were not collected in the survey.

Given the high prevalence of anaemia among pregnant women, we recommend that this public health problem be addressed urgently. While the measures needed to address this public health issue should be a national one, control efforts should be intensified in the North-central and south south, whose pregnant women had higher odds of anaemia. Girl-child education through intersectoral collaboration (Ministry of Education, Ministry of Women Affairs, Ministry of Information) must continue to be on the front burner as educated girls become educated women who can make better informed decisions on their pregnancy and follow through health workers' recommendation for improved health status. Educated women are also more likely to be economically empowered and are more likely to have better care-seeking attitude which will in turn lead to prevention, early detection, and treatment of anaemia with an attendant improvement in pregnancy outcome for both mother and baby. In addition to these measures, social programs for pregnant women that is especially targeted at women who are not in a marital union (single/separated, divorced, widowed) to provide them with the social and economic resources to be able to go through pregnancy.

With the unexpected higher risk of anaemia in pregnancy among women in the Northcentral and Southsouth compared to women in the Northwest, despite the seemingly better social determinants of health in the former two regions, future research could help identify those factors that put women in those two regions at a higher risk. Furthermore, future research could adjust for other variables like bacterial infections, nutritional intake, malaria, and hookworm infestation, which are especially important factors that could predispose to anaemia.

In conclusion, clinicians and policy makers must recognize that women residing in the Northcentral and Southsouth zones are particularly at risk for anaemia in pregnancy and should be identified as high-risk groups with particular attention paid to them during antenatal care. Other risk factors like not being in a union could be addressed by providing socioeconomic help (for example, free health care, free supplements, nutrient-rich foods) to this group during pregnancy. To address low educational attainment as a risk factor, girl child education must be made a national priority.

## Data Availability

The 2018 Nigeria DHS dataset can be obtained from the DHS program (ICF, 530 Gaither Road, Suite 500, Rockville, MD 20850, USA) or from the National Population Commission, Abuja, Nigeria or from The DHS Program - Available Datasets.
